# Association of Aggression and Non-Suicidal Self Injury: A School-Based Sample of Adolescents

**DOI:** 10.1371/journal.pone.0078149

**Published:** 2013-10-30

**Authors:** Jie Tang, Ying Ma, Yong Guo, Niman Isse Ahmed, Yizhen Yu, Jiaji Wang

**Affiliations:** 1 School of Public Health, Guangzhou Medical University, Guangzhou, Guangdong, China; 2 Guangzhou Women and Children’s Medical Center, Guangzhou, Guangdong, China; 3 Department of Child, Adolescence & Woman Health Care, School of Public Health, Tongji Medical College, Huazhong University of Science & Technology, Wuhan, Hubei, China; The University of Queensland, Australia

## Abstract

**Purpose:**

Non-suicidal self-injury (NSSI) in adolescent has drawn increasing attention because it is associated with subsequent depression, drug abuse, anxiety disorders, and suicide. In the present study, we aimed to estimate the prevalence of non-suicidal self-injury (NSSI) in a school-based sample of Chinese adolescents and to explore the association between aggression and NSSI.

**Methods:**

This study was part of a nationwide study on aggression among adolescents in urban areas of China. A sample of 2907 school students including 1436 boys and 1471 girls were randomly selected in Guangdong Province, with their age ranging from 10 to 18 years old. NSSI, aggression, emotional management and other factors were measured by self-administrated questionnaire. Multinomial logistic regression was used to estimate the association between aggression and NSSI, after adjustment for participants’ emotional management, and other potential confounding variables.

**Results:**

The one year self-reported prevalence of NSSI was 33.6%. Of them, 21.7% engaged in ‘minor NSSI’, 11.9% in ‘moderate/severe NSSI’. 96.9% of self-injuries engaged in one to five different types of NSSI in the past year. Hostility, verbal and indirect aggression was significantly associated with self-reported NSSI after adjusting for other potential factors both in ‘minor NSSI’ and ‘moderate/severe NSSI’. Hostility, verbal and indirect aggression was significantly associated with greater risk of ‘minor NSSI’ and ‘moderate/severe NSSI’ in those who had poor emotional management ability.

**Conclusion:**

These findings highlight a high prevalence of NSSI and indicate the importance of hostility, verbal and indirect aggression as potentially risk factor for NSSI among Chinese adolescents.

## Introduction

Non-suicidal self injury (NSSI) is not an illness but a behavior often enacted by individuals with the intention of physically harming themselves [1]. Most common methods of self harm include self-cutting, self-hitting, self-poisoning and overdosing [2]. Recently, NSSI among adolescents has attracted increasing attention and concern around the world, because NSSI tends to first occur during adolescence is associated with depression, drug abuse, anxiety disorders, [3], and suicide [4], [5].

In the past two decades, there was an upward trend in NSSI prevalence in adolescents, although the reported prevalence of NSSI varied across countries. Hawton et al. conducted an anonymous self-report survey in 41 schools in England and they reported a lifetime prevalence of 13.2% among 15∼16 year olds in 2000/2001 [5]. A study conducted in Sweden reported a lifetime prevalence of 17.1% among 17 years olds [6]. In the United States, lifetime prevalence of NSSI generally ranged from 12%∼20.2% in secondary school children and 12%∼20% in late adolescent and young adults [7], [8]. In one of the largest epidemiological studies of adolescents to date in the U.S. (n = 61767), Taliaferro and colleagues reported a 12-month prevalence of 7.3% for NSSI [9]. High prevalence of NSSI has also been found in China. Sun et al. reported a prevalence of 22.3% among junior high and high school students in rural Anhui [10]. Daniel et al. conducted a survey among 3328 secondary school students in Hong Kong and they reported that 32.7% of the students had at least one form of NSSI during the previous 1 year [11]. Many factors contributed to those inconsistencies prevalence of NSSI, such as sampling, assessment instruments, time frames, and different classification system for NSSI, and etc, while there is a consensus that adolescence is a risky period in which NSSI may occur [12].

NSSI in adolescents inflicts actual harm and repetitive and chronic NSSI may increase the risk of suicide [13]. Therefore, effective prevention approaches are needed. Previous studies have been conducted to identify risk and protective factors for adolescents at different levels. At the individual level, gender was a predictor of NSSI; girls had significantly higher NSSI prevalence than boys [14]. At family level, a number of studies suggested that low family economic status [15], parental marriage status [16], and severe family dysfunction were associated with adolescent NSSI [17]. With regard to the personal characteristics, it has been shown that negative emotion and self-derogation were associated with adolescent NSSI [18]. NSSI was also related to individual psychological well-being [18].

Aggression may be an important risk factor for NSSI. First of all, psychologically speaking, aggression is an important diathesis part of self-injury behavior according to the stress-diathesis model proposed by John Mann. Individuals with this diathesis might be likely to experience more self-injury feeling and thought, and this diathesis was more important than the psychiatric illness in predicting suicidal behaviors [19]. On the other hand, aggression and NSSI may have a common basis in pathophysiology, namely, the abnormal serotonergic system [20], [21]. Surprisingly, there is rare study has explored the association between aggression and NSSI. Studies on exploring the association of aggression and suicidal behaviors revealed that aggression may act as a predictor of future suicide and elevate the risk for suicidal behaviors. For example, Keilp and his colleagues found that aggressiveness was the most important predictor of suicidal behavior when stratifying by borderline personality disorder [22]. Zhang and her colleagues found that hostility and physical aggression and trait anger may predict suicidal behavior among adolescents [23].

Both NSSI and suicidal behaviors are self-harm behaviors which may have the same risk factors, though previous study suggested that NSSI acted as a method of acquiring ability to enact suicidal behaviors [24]. Considering the facts mention above, the present study attempted to examine the correlation of NSSI and aggression among Chinese adolescents based on a larger and representative sample of middle school students, as well as to re-estimate the prevalence of NSSI among youth. Based on the literature review, we hypothesized that aggression may be associated with NSSI, even after taking into account some potential variables.

## Methods

### Study Population

Data were collected as part of the Nationwide Study on Aggression among adolescents in urban of China, which initiated in 2010. The overall study included nationwide representative samples of approximately 500 students at each grade from 7 to 12 in each of five provinces: Guangdong, Anhui, Hubei, Heilongjiang, and Yunnan, and detailed sampling methods were described elsewhere [23]. Data analyzed in the present study were from Guangdong students, because the questionnaires for NSSI were only collected in Guangdong Province. Overall, 3161 students were recruited and a consent letter was sent to their parents or their guardians. Of these study subjects, 43 refused to participate in the study and 32 were absent from the school at time of survey, therefore, the survey was completed by 3096 students of grade 7 to 12. By an initial screening based on the completeness of the questionnaires, 179 students were excluded due to incomplete questionnaire. Thus, the final study population consisted 2907 students, 1436 boys and 1471 girls with a mean age of 15.4 (SD = 1.8) years, ranging from 10 to 18 years. The actual response rate of the participants was 92.0% (2907/3161).

A written informed consent was obtained from the parents or the guardians of each participant. The research protocol, including the questionnaires, was approved by the targeted schools and the Medical Ethics Committee of Tongji Medical College, Huazhong University of Science and Technology and Guangdong Education Committee.

Data collection was carried out by a group of trained study staffs, who explained the purpose and procedures of the study. The self-administered survey was anonymous, and it was completed in classrooms during a 45- to 60-minute period. Before the participants completed the self-administered questionnaire, they had been told that the questionnaire did not represent a test and that there were no correct or incorrect answers and we had promised each participant that their answers will be kept confidential and the data will only be used for scientific research. The same written announcements were printed in the front of the questionnaire. The emphasis was placed on answering the questions honestly and accurately.

### Instruments

#### Non-suicidal self-injury

The Functional Assessment of Self-Mutilation (FASM) was designed to assess the methods, frequency and function of self-reported NSSI for the participants over the previous 12 months [25]. It presented in checklist format, of which respondents were asked whether they purposefully engaged in each of 10 different NSSI behaviors, if so, the frequency of occurrence was obtained. A principal components analysis of the 10 behaviors yielded two factors. The first factors included items considered more clinically severe in nature, donated as ‘moderate/severe NSSI’: cutting/carving, burning, self- tattooing, scraping, and erasing skin (i.e. using an eraser to rub skin to the point of burning and bleeding). The second factor contains less severe behaviors, denoted as ‘minor NSSI’: hitting self, pulling hair, biting self inserting objects under nails or skin, picking areas to draw blood and picking at a wound. Participants were also asked whether any of these behaviors was a suicide attempt. The FASM also consists of 22 statements assessing motivations for NSSI, which was not measured in the present study. The FASM has been demonstrated acceptable psychometric properties within adolescent samples, yielding adequate internal consistency (α = 0.65∼0.66) for minor and moderate/severe NSSI [26].

#### Aggression

The Chinese version of Buss and Warren’s Aggression Questionnaire (BWAQ) was administered to assess aggression [27]. It is a self-rating questionnaire consisting of 34 items. Each item was answered on a 5-point Likert scale ranging from 1 (not at all like me) to 5 (completely like me). The questionnaire measured five constructs related aggression: physical aggression (PHY), verbal aggression (VER), anger (ANG), hostility (HOS), and indirect aggression (IND), the higher scores of each construct reflecting greater aggression. According to Buss and Warren’s interpretation of aggression, the related aggression scores (PHY, VER, ANG, HOS and IND) were group into seven categories: very low, low, low average, average, high average, high, and very high, as described in details elsewhere [28]. In the present study, students who had ‘high average’ or ‘high’ or ‘very high’ score were classified as having related aggression. The BWAQ has been reported to have good psychometric properties (internal reliability: physical aggression = 0.81; verbal aggression = 0.71; anger = 0.64; hostility = 0.61; indirect aggression = 0.62) [29]. The internal consistency as reflected by overall Cronbach coefficient alpha of these 34 items in our study was 0.88, and the internal consistency for each subscale was 0.75, 0.51, 0.61, 0.69, and 0.58, respectively.

#### Emotional management scale

The Emotional Intelligence Inventory (EII) included five subscales which measured five constructs related emotion: cognitive self-emotional ability, cognitive other’s emotional ability, emotional management ability, self-motivation ability, interpersonal relationship management ability. Each item was answered on a 4-point Likert scale ranging from 1 (always like this) to 4 (never like this), the higher scores of each construct reflecting greater ability for emotion related [30]. In the present study, only the emotional management subscale (include 4 items) was used to assess the emotional management ability of the participants. The subscale had good internal consistency (α = 0.75). In order to better understand the effect of emotional management on association between NSSI and aggression, the scale scores were group into three levels: poor, average and good. Score cutoffs were as following: poor<1SD, 1SD≤average≤1SD, good >1SD [30].

#### Other risk factors

A wide range of social, family and school factors were measured by an additional questionnaire. They were perceived social atmosphere (good/fair/poor), parents’ highest education, family structure (extended or nuclear family/step family/single-parent family/grandparent family/others), one child family (yes/no), accordance of parenting styles (yes/no), perceived school atmosphere (good/fair/poor), perceived relationship with teachers and classmates (good/fair/poor), academic performance (good/fair/poor), numbers of close friends (none/one and above) and satisfaction of appearance (satisfied/fair/unsatisfied). The questionnaire was pretested among 48 students to ensure content and language was appropriate for the study population. Additionally, consistency test was examined. Participants were surveyed at two time point, ten days apart, with the participants using self-assigned identification numbers so surveys could be linked across time points. The Kappa value between surveys was up to 0.89.

### Statistical Analysis

NSSI and Aggression statistics (frequency and percentage) were calculated to reflect the epidemiological characteristics of the study sample. Chi-square tests and *t*-tests were used to compare frequencies and continuous data, respectively. As the categorical dependent outcome has more than two levels, we estimated the association between aggression and NSSI using a multinomial logistic regression. We reported both unadjusted and adjusted odds ratios and 95% confidence intervals for the potentially confounding effects of aggression, emotional management, and demographic variables. Demographic variables included gender, age, family and school environment and so on. Significance level was set at 0.05, and all tests were two sided. Statistical analyses were conducted using SPSS for Windows 15.0 (SPSS Inc., Chicago, IL).

## Results

### Overview

A total of 2907 students (1436 boys and 1471 girls) were included in this study. The demographic characteristics and related risk behaviors of boys and girls are summarized in Table 1. Self-reported 1-year prevalence of NSSI was 33.6%, with 21.7% and 11.9% students engaged in ‘minor’ NSSI and ‘moderate/sever’ NSSI, respectively. Girls were more likely to be engaged in NSSI than boys (both ‘minor’ and ‘moderate/sever’ NSSI) ( *χ* ^2^ = 9.408, *p* = 0.009). No significance on indirect aggression and hostility was found between boys and girls (*χ* ^2^ = 0.002, *p* = 0.966; *χ* ^2^ = 1.585, *p* = 0.208); while significance on physical aggression, verbal aggression, anger and hostility were found (*χ* ^2^ = 42.911, *p*<0.001; *χ* ^2^ = 18.012, *p*<0.001; *χ* ^2^ = 7.739, *p* = 0.005). Boys were likely to have a greater ability of emotional management (*t* = 4.617, *p*<0.001).

**Table 1:** Demographic characteristics and related behaviors of the study population.

	Boys(N = 1436)	Girls(N = 1471)	Total (N = 2907)
Age (Mean, SD)	15.4±1.78	15.4±1.79	15.4±1.78
EM (Mean, SD)	11.3±2.44	10.9±2.39	11.1±2.42
Aggression			
PHY (n, %)	400(27.9)	260(17.7)	660(22.7)
VER (n, %)	350(24.4)	264(17.9)	614(21.1)
ANG (n, %)	354(24.7)	430(29.2)	784(27.0)
HOS (n, %)	323(22.5)	360(24.5)	683(23.5)
IND (n, %)	327(22.8)	334(22.7)	661(22.7)
NSSI (n, %)			
No-NSSI	992(69.1)	939(63.8)	1931(66.4)
Minor NSSI^a^	292(20.3)	339(23.0)	631(21.7)
Moderate/severe NSSI^b^	152(10.6)	193(13.1)	345(11.9)

Note: EM = Emotional Management; NSSI = Non-suicidal self-injury; PHY = physical aggression; VER = verbal aggression; ANG = anger; HOS = hostility; IND = indirect aggression.

a Minor NSSI including hitting self, pulling hair, biting self, inserting objects under nails or skin, picking at a wound.

b Moderate/severe NSSI included cutting/carving, burning, self-tattooing, scraping, and erasing skin.

### Descriptive Characteristics of NSSI Among Adolescent

Table 2 shows the frequency and prevalence of each NSSI method. Of the 33.6% of the overall sample who engaged in one or more of the ten NSSI behaviors in the past year, 96.9% of the self-injuries reported engaging in one to five different types of NSSI; the mean number of types of NSSI performed was 2.2 (SD = 1.4, median = 2.0, mode = 1.0, range = 1–10). Hitting self, biting self, pulling hair out, and picking at wound were the most frequent NSSI behaviors in the study population.

**Table 2:** Descriptive characteristics of NSSI within the past year of the study population.

	NSSI frequencies (n = 2907)							
	0		1∼5		6∼10		>10	
Type of NSSI	n	%	n	%	n	%	n	%
**Minor NSSI**								
Hit self	2366	81.4	411	14.1	107	3.7	23	0.8
Pulled hair out	2477	85.2	295	10.1	120	4.1	14	0.5
Inserted objectives under nail or skin	2664	91.6	176	6.1	63	2.2	4	0.1
Bit self	2467	84.9	318	10.9	109	3.7	13	0.4
Pick at wound	2507	86.2	293	10.1	104	3.6	3	0.1
**Moderate/severe NSSI**								
Cut/carved on skin	2718	93.5	160	5.5	24	0.8	5	0.2
Self-tattoo	2729	93.9	143	4.9	35	1.1	0	0.0
Burned skin	2874	98.9	21	0.7	12	0.4	0	0.0
Scraping skin	2791	96.0	100	3.4	16	0	0	0.0
Erased skin	2887	99.3	14	0.5	6	0.2	0	0.0

### Is there Association between Aggression and NSSI?

Gender difference on occurrence rates of NSSI was existed, thus we examined the association of NSSI and aggression stratified by gender. The results are presented in Table 3. Self reported NSSI were significantly associated with aggression (including PHY, VER, ANG, HOS, and IND) in both boys and girls. Subjects who reported NSSI during the past 12 months before the investigation were more likely to experience aggression. The odds ratios for NSSI in boys were 1.51, 1.80, 1.57, 2.33, and 1.73 respectively, and in girls were 1.79, 1.91, 1.94, 2.63, and 1.95 respectively. Those associations were not significantly differentiated by gender. Therefore, we included both sexes in the following analysis.

**Table 3:** Numbers and column percents for aggression with reported NSSI stratified by gender.

		Boys(N = 1436)					Girls(N = 1471)				
		Non-NSSI	Minor NSSI	Moderate NSSI	*χ* ^2^	*p*	Non-NSSI	Minor NSSI	Moderate NSSI	*χ* ^2^	*p*
PHY	YES	250(62.5)	99(24.8)	51(12.8)	11.248	0.004	136(52.3)	73(28.1)	51(19.6)	20.197	0.000
	No	742(71.6)	193(18.6)	101(9.7)			803(66.3)	266(22.0)	142(11.7)		
VER	YES	207(59.1)	91(26.0)	52(14.9)	21.902	0.000	135(51.1)	84(31.8)	45(17.0)	22.649	0.000
	No	785(72.3)	201(18.5)	100(9.2)			804(66.6)	255(21.1)	148(12.3)		
ANG	YES	218(61.6)	74(20.9)	62(17.5)	25.210	0.000	227(52.8)	109(25.3)	94(21.9)	48.385	0.000
	No	774(71.5)	218(20.1)	90(8.3)			712(68.4)	230(22.1)	99(9.5)		
HOS	YES	175(54.2)	94(29.1)	54(16.7)	43.961	0.000	167(46.4)	112(31.1)	81(22.5)	68.142	0.000
	No	817(73.4)	198(17.8)	98(8.8)			772(69.5)	227(20.4)	112(10.1)		
IND	YES	195(59.6)	80(24.5)	52(10.6)	20.333	0.000	172(51.5)	87(26.0)	75(22.5)	40.692	0.000
	No	797(71.9)	212(19.1)	100(9.0)			767(67.5)	252(22.2)	118(10.4)		

### Can Confounding Variables Explain the Association between NSSI and Aggression?

To examine those associations in a multivariate context, multinomial logistic regression analysis was conducted, in which self-reported ‘minor NSSI’ and ‘moderate/severe NSSI’ were regressed on aggression, along with controlling for other potential confounding variables. Results are showed in Table 4. Model I showed that VER, HOS and IND were significantly associated with both ‘minor NSSI’ and ‘moderate/severe NSSI’ after controlling for gender, satisfaction of appearance, and other demographic characteristics (OR_VER_ = 1.54/1.75, OR_HOS_ = 2.09/1.93, OR_IND_ = 1.21/1.63). However, no significance between PYH, ANG and NSSI was found.

**Table 4:** Multinomial Logistic regression models for predicting NSSI.

Group^a^		Model I	Model II	Model III	Model IV
		OR (95% CI)	OR (95% CI)	OR (95% CI)	OR (95% CI)
Minor NSSI	VER (yes vs. no)	1.54^#^ (1.24∼1.92)	1.58^#^ (1.26∼1.99)	1.73^#^ (1.36∼2.19)	1.73(1.37∼2.19)^#^
	HOS (yes vs. no)	2.09^#^ (1.69∼2.58)	1.92^#^ (1.52∼2.44)	2.16^#^ (1.69∼2.77)	2.16(1.68∼2.76)^#^
	IND (yes vs. no)	1.21 (1.06∼1.35)	1.17^#^ (1.08∼1.25)	1.16^#^ (1.06∼1.25)	1.20(1.08∼1.32)^#^
	One child (yes vs. no)	0.75^#^ (0.64∼0.90)	0.75^#^ (0.62∼0.91)	0.74^#^ (0.61∼0.90)	0.74(0.61∼0.90)^#^
	Gender (female vs. male)	1.23^#^ (1.02∼1.48)	1.24^#^ (1.02∼1.50)	1.26^#^ (1.04∼1.52)	1.23(1.02∼1.42)^#^
	Satisfaction of appearance (fair vs. poor)	0.76 (0.52∼1.10)	0.77 (0.53∼1.12)	0.76(0.52∼1.10)^#^	0.75(0.52∼1.10)^#^
	Satisfaction of appearance (good vs. poor)	0.69^#^ (0.47∼0.98)	0.71 (0.48∼1.03)	0.69(0.47∼0.99)^#^	0.69(0.47∼0.89)^#^
	EM (fair vs. poor)	–	0.73^#^ (0.57∼0.95)	0.83(0.76∼0.90)^#^	0.69(0.55∼0.86)^#^
	EM (good vs. poor)	–	0.58^#^ (0.40∼0.83)	0.73(0.64∼0.82)^#^	0.45(0.32∼0.62)^#^
	Aggression*EII (fair)	–	–	0.56(0.39∼0.79)^#^	–
	Aggression*EII (good)	–	–	0.39(0.24∼0.50)	–
	Aggression*EII*Gender (fair)	–	–	–	0.81(0.64∼1.09)
	Aggression*EII*Gender s (good)	–	–	–	0.21(0.04∼1.32)
	Constant	−2.45	−0.15	−0.91	−0.17
Moderate/severe NSSI	VER (yes vs. no)	1.75^#^ (1.33∼2.30)	1.63^#^ (1.23∼2.15)	1.60^#^ (1.18∼2.15)	1.61(1.20∼2.17)^#^
	HOS (yes vs. no)	1.93^#^ (1.45∼2.58)	1.72^#^ (1.29∼2.31)	1.76^#^ (1.29∼2.40)	1.78(1.30∼2.42)^#^
	IND (yes vs. no)	1.63^#^ (1.34∼2.15)	1.54^#^ (1.17∼2.04)	1.57^#^ (1.16∼2.11)	1.59(1.18∼2.14)^#^
	One child (yes vs. no)	0.77 (0.60∼0.98)	0.80(0.63∼1.03)	0.80 (0.62∼1.03)	0.80(0.62∼1.03)
	Gender (female vs. male)	1.30^#^ (1.02∼1.66)	1.23 (0.96∼1.23)	1.24^#^ (0.97∼1.58)	1.27(0.98∼1.63)^#^
	Satisfaction of appearance (fair vs. poor)	0.65 (0.42∼1.01)	0.66 (0.42∼1.02)	0.66(0.43∼1.02)	0.66(0.43∼1.03)^#^
	Satisfaction of appearance (good vs. poor)	0.56^#^ (0.36∼0.86)	0.58^#^ (0.38∼0.90)	0.59^#^(0.38∼0.92)	0.59(0.38∼0.92)^#^
	EM (fair vs. poor)	–	0.58^#^ (0.43∼0.78)	0.62^#^(0.43∼0.88)	0.62(0.44∼0.88)^#^
	EM (good vs. poor)	–	0.19^#^ (0.11∼0.35)	0.21^#^ (0.11∼0.40)	0.22 (0.12∼0.42)^#^
	Aggression*EII (fair)	–	–	0.86^#^(0.73∼0.99)	–
	Aggression*EII (good)	–	–	0.84^#^ (0.77∼0.91)	–
	Aggression*EII*Gender (fair)	–	–	–	0.89(0.69∼1.15)
	Aggression*EII*Gender (good)	–	–	–	0.68(0.23∼2.06)
	Constant	−1.39	−1.17	−1.27	−1.32

Note: a = The reference category is: no NSSI; CI = Confidence Interval, ^#^ *p<*0.05.

### Can Emotional Management Explain Association between NSSI and Aggression?

A greater ability of emotional management may decrease the likelihood that the adolescents will engage in NSSI. Therefore, we examined whether emotional management could change the association between NSSI and aggression (Table 4, Model II). However, the association between VER, HOS, IND and NSSI remained after adjustment for emotional management and other demographic characteristics in both of the two groups (OR_VER_ = 1.58/1.63, OR_HOS_ = 1.92/1.72, OR_IND_ = 1.17/1.54).

The interaction of emotional management with aggression may effect on the association between NSSI and aggression, Therefore, the interaction of emotional management and aggression was added separately to the regression equation (Table 4, Model III). The coefficient for the interaction of emotional management (average level vs. poor level) and aggression was significant and negative in both ‘minor NSSI’ (β = −0.58, SE = 0.07) and ‘moderate/severe NSSI’ (β = −0.15, SE = 0.09), indicating that aggression was associated with a high risk of NSSI among those who had a poorer ability of emotional management. Odds ratios calculated from the coefficients in Model III indicated that have aggression on VER, HOS and IND were associated with a greater risk of self-reported ‘minor NSSI’ and ‘moderate/severe NSSI’ among those who has a poor ability of emotional management (OR_VER_ = 3.09/1.86, OR_HOS_ = 3.86/2.04, OR_IND_ = 2.07/1.82), while they were associated with relative lower risk among those who had an average emotional ability (OR_VER_ = 0.97/1.38, OR_HOS_ = 1.21/1.51, OR_IND_ = 0.65/1.35).

Additional models estimated whether the association between aggression and NSSI persisted after adjustment for other confounding variables and interactions. No interaction terms estimated in this model achieved statistical significance level at 0.05 (Model IV).

## Discussion

The present study enhanced our understanding of NSSI and explored the association of NSSI with aggression among Chinese adolescents based on a large sample of school students. First, there is evidence regarding the association between NSSI and aggression, indicating that hostility, verbal aggression and indirect aggression may predict NSSI among adolescents, even after taking emotional management and demographic characteristics into account. Second, this study reported that approximately 33.6% of the sample reported having engaged in NSSI during the past one year in Guangdong Province.

NSSI was not usually considered as an illness and this made people pay less attention to this behavior, which may partly explain the rising prevalence of NSSI all around the world [31]. Prior studies about lifetime prevalence of NSSI suggested that 15% to 20% of community-based youth have engaged in NSSI in the west, and approximately 28% of those reported moderate to severe behavior such as cutting, burning, scraping or erasing the skin [32]. In the present study, however, the prevalence of NSSI was higher than that reported in the west. One of reasons for this difference may be that we did not study rural areas, where prevalence of NSSI is lower. A study conducted in rural Anhui Province, China, reported that the prevalence of NSSI was 22.3% [10]. As a result, our finding may overestimate the prevalence of NSSI among adolescent as a whole. Second, NSSI is prone to occur during 12∼15 year old adolescents [3], in this study, the participants were junior high or high school students, and elementary school students were not surveyed. Therefore, differences in the age distribution of study samples may partly account for this difference and the estimate of NSSI could not be directly compared with the data from the West. Moreover, self-reported NSSI prevalence in the present study was differed from the previous studies conducted in China [24], [33]. Our findings provide important information for making professionals and other societies aware that NSSI is becoming a rising serious health problem for adolescents and thus needs urgent interventions.

Previous studies on risk factors of NSSI have focused on psychosocial factors, such as socioeconomic status [34], family dysfunction [35], cognition [36], and so on. Despite the burgeoning interest in NSSI and increasing number of studies on determinants, remarkably little is known about the association between aggression and NSSI at critical developmental periods during adolescence. In the present study, hostility, verbal and indirect aggressions were associated with NSSI after controlling for emotional management, demographic and family characteristics.

Possible explanations for the finding are as follows. First, according to the construction of BWAQ, hostility, the cognitive component of aggression, includes ‘resentment, social isolation, and paranoia’ [37]. Hostility is not only associated with distrust of others, vulnerability to stress, poor coping and frequent negative affect, but also associated with anxiety and depression [38], and all of those competence mentioned above are commonly known as risk factors accounting for NSSI [34], [35], [36].

Second, according Nock’s theory that individuals with less positive emotionality differentiation will be more likely to engage in NSSI [39], and aggression including verbal aggression, indirect aggression, hostility, physical aggression, and anger can be defined as less positive emotional tendencies which developed from an complicated factors. However, physical aggression and anger were not significantly associated with NSSI after controlling for demographic characteristics and other potential variables. This was inconsistent with similar studies. Zhang and her colleagues’ study suggested that physical aggression and trait anger is significant associated with suicidal behavior, and this difference confirmed that the aetiological profiles NSSI and suicidal behaviors are not always the same [23]. Additionally, direct aggression (including physical aggression and verbal aggression) and indirect aggression may change with age. Direct aggression of physical or verbal is common in young children [40], and as children age, physical aggression tends to decrease and verbal aggression tends to increase [41]. As children develop in their social understanding, they become more capable of managing indirect forms of aggression.

Third, previous studies indicated that aggression may be a way to regulate one’s emotions. However, it may not be effective in all types of aggression [42], [43]. When someone’s aggression is not effective to regulate emotions, NSSI may be commonly carried out among youth [44], especially in youth with poor emotional management ability. In the present study, verbal aggression, hostility and indirect aggression were associated with a high risk of ‘minor NSSI’ or ‘moderate/severe NSSI’ among those who had a poorer ability of emotional management, while were associated with relatively lower risk among those who had an average emotional ability. Adolescents with greater emotional management ability always develop skills for controlling ones’ emotion, and make effective behavioral choices to regulate one’s negative emotion. A high sense of self-determination allows an individual to think for oneself and to take action consistent with that thought [45].

The present study must be interpreted in light of several limitations. Firstly, although the study achieved a relatively large sample size, the participants only include urban students, which may limit the generalizability of our findings. Secondly, these data were based on retrospective self-reports, which may introduce potential problems with overestimate or underestimate of NSSI. Moreover, there were only ten items on NSSI which did not capture all acts of deliberate self-harm (e.g. drug overdose) [46]. Nonetheless, the self-reported NSSI in our study were similar to those findings in another independent study conducted based on school students of the same age group [11]. Thirdly, the present study did not include all the factors when assessing the association between aggression and NSSI, those included subjects’ psychological factors (anxiety, distress, depression, etc), family function (family neglect, family maltreated, etc), impulsivity and so on, which may lead to an underestimate of strength of the association. Therefore, the investigation of these and other factors remains key directions for future research.

Lastly, this study was cross-sectional, and causal relationship between aggression and NSSI should be inferred with caution and cannot be established on these data. Therefore, the findings need to be validated in a prospective cohort study.
